# ERK contributes to B cell receptor-induced cell spreading in the A20 mouse B cell line

**DOI:** 10.17912/micropub.biology.000665

**Published:** 2022-11-23

**Authors:** Victoria Peters, Nikola Deretic, Kate Choi, Michael R Gold

**Affiliations:** 1 Department of Microbiology & Immunology and the Life Sciences Institute, University of British Columbia, Vancouver, Canada

## Abstract

B cells provide protective immunity by secreting antibodies. When a B cell encounters its specific antigen, B-cell receptor (BCR) signaling initiates actin remodeling. This allows B cells to spread on antigen-bearing surfaces and find more antigen, which increases BCR signaling and facilitates B cell activation. The BCR activates multiple signaling pathways that target actin-regulatory proteins. Although the extracellular signal-regulated kinases ERK1 and ERK2 regulate actin-dependent processes in adherent cells, their role in BCR-induced actin remodeling had not been investigated. Here, we show that targeting ERK with chemical inhibitors or siRNA inhibits BCR-induced spreading in a murine B cell line.

**Figure 1. ERK activity is important for BCR-induced spreading:**
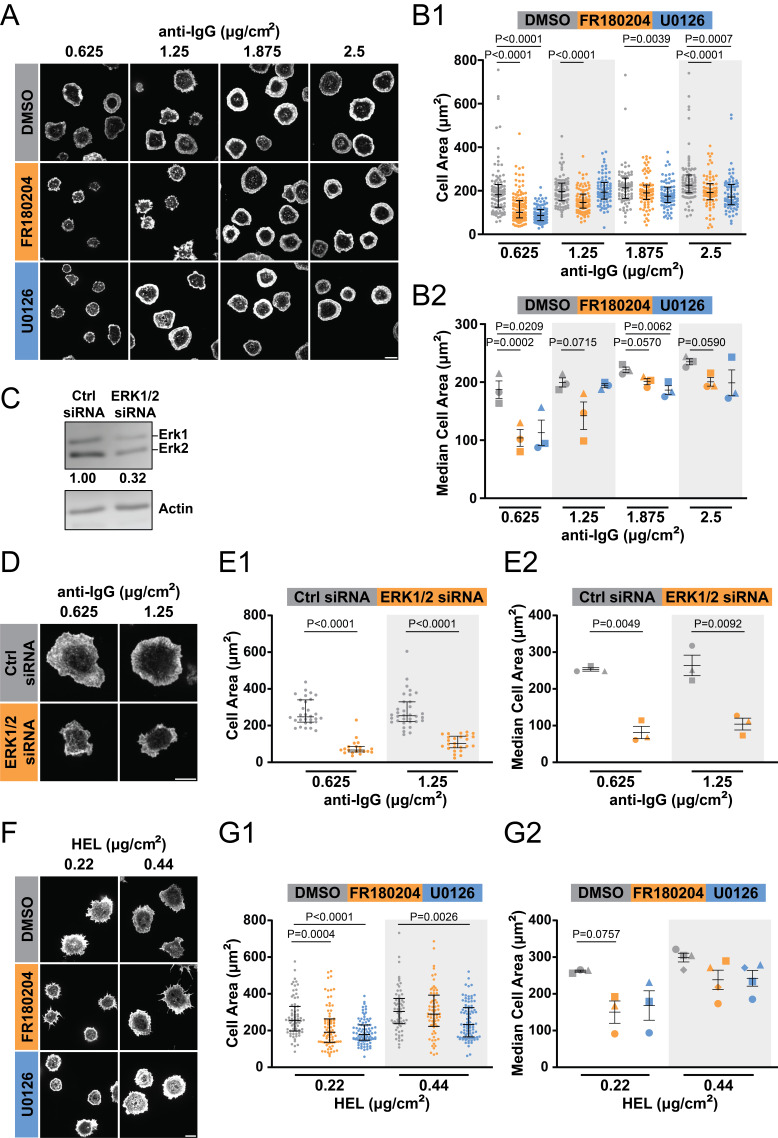
**(A,B)** A20 cells were pre-treated with 30 µM of the ERK inhibitor FR180204 or the MEK inhibitor U0126 for 1 hr and then allowed to spread for 30 min on coverslips coated with the indicated amounts of goat anti-mouse IgG. The cells were stained with rhodamine-phalloidin to visualize F-actin and the peripheral F-actin ring was used to define the outer edges of the cells and quantify cell areas. Representative images are shown in (A). Data from a representative experiment are shown in (B1). Each dot is one cell and the median and interquartile ranges are shown. The graph in (B2) shows compiled data from 3 independent experiments with n = 57-134 cells per condition. Each symbol is an individual experiment and the data are presented as the mean + SEM for the median values from the 3 experiments. **(C-E)** A20 D1.3 cells were transfected with either control non-targeting siRNA or a combination of siRNAs directed against ERK1 and ERK2. In (C), cell lysates were analyzed by immunoblotting with an ERK1/2 antibody or with an actin antibody (loading control). ERK1 + ERK2 levels relative to the control siRNA sample are shown beneath the ERK blot. The siRNA-transfected cells were allowed to spread for 30 min on coverslips coated with the indicated amounts of goat anti-mouse IgG. Representative images are shown (D) along with data from a representative experiment (E1) and compiled data from 3 independent experiments (E2) with n = 20-34 cells per condition. **(F,G)** A20 D1.3 cells were pre-treated with 30 µM of the ERK inhibitor FR180204 or the MEK inhibitor U0126 for 1 hr and then allowed to spread for 30 min on coverslips coated with 0.22 µg/cm ^2^ or 0.44 µg/cm ^2^ HEL. Representative images are shown (F) along with data from a representative experiment (G1) and compiled data from 3 independent experiments (G2) with n = 40-87 cells per condition. All scale bars are 10 µm. P-values were calculated using the Mann Whitney U test (panels B1, E1, G1) or Student’s paired t-test (panels B2, E2, G2). Only P-values <0.1 are shown.

## Description

When B cells encounter immobilized antigens, or antigens that are tethered to planar lipid bilayers, they undergo radial spreading that is driven by the formation of large lamellipodia (Freeman et al., 2011). As the cells spread, a peripheral ring of F-actin forms, concomitant with clearance of F-actin from the center of the substrate contact site (Bolger-Munro et al., 2019). Branched actin polymerization nucleated by the Arp2/3 complex drives the formation of this peripheral actin ring and exerts outward force on the plasma membrane that drives cell spreading (Pollard and Borisy, 2003). This spreading response is thought to mimic the initial steps that occur when B cells encounter antigens that are displayed on the surface of antigen-presenting cells (APCs), resulting in the formation of an immune synapse (Harwood and Batista, 2010; Abraham et al., 2016).

The binding of antigens to the B cell antigen receptor (BCR) initiates the activation of multiple signaling pathways (Abraham et al., 2016), including Ras-dependent activation of the ERK1 and ERK2 kinases (Gold et al., 1992; Tordai et al., 1994; Sutherland et al., 1996). Although ERK promotes membrane protrusion and cell motility in a variety of adherent cell types (Mendoza et al., 2015; Tanimura and Takeda, 2017; Hirata and Kiyokawa, 2019; Lavoie et al., 2020), its role in actin-mediated processes in lymphocytes has not been studied.

To assess the role of ERK in BCR-induced cell spreading we used the IgG+ A20 murine B-lymphoma cell line and its derivative, the A20/D1.3 cell line. A20 cells have been widely used to study B cell spreading on surfaces displaying anti-IgG antibodies. Anti-Ig antibodies bind to the constant region of the membrane Ig subunit of the BCR and initiate signaling by clustering BCRs. In addition to its endogenous BCR, A20/D1.3 cells express a transfected hen egg lysozyme (HEL)-specific BCR derived from the D1.3 monoclonal antibody (Batista and Neuberger, 1998). This allows us to study B-cell spreading initiated by the binding of HEL to the antigen-binding site of the BCR.

To assess the role of ERK in B cell spreading we pre-treated A20 or A20/D1.3 cells with either FR180204, a selective inhibitor of ERK activity (Ohori et al., 2005), or U0126, an effective inhibitor of MEK1 and MEK2 (Favata et al., 1998), the kinases that phosphorylate and activate ERK1 and ERK2 (Lavoie et al., 2020). U0126 has been used to inhibit the MEK/ERK pathway in B cells (Richards et al., 2001). Figure 1A and 1B show that pre-treating A20 cells with 30 µM FR180204 for 1 hr reduced the ability of B cells to spread on immobilized anti-IgG antibodies. Greater inhibition was observed when the coverslips were coated with 0.625 µg/cm ^2^ than when the they were coated with 1.25 µg/cm ^2^ . At higher anti-IgG densities, e.g., 2.5 µg/cm ^2^ , inhibiting ERK did not have a significant impact on BCR-induced spreading. Similarly, the MEK inhibitor U0126 significantly reduced the spreading of A20 cells on coverslips coated with 0.625 µg/cm ^2^ anti-IgG but had much less effect at higher anti-IgG densities. This suggests that activation of the MEK/ERK pathway is more important for B cell spreading when the antigen density is low, as it might be on the surface of APCs that capture soluble antigens.

To validate the role of ERK in BCR-induced spreading we used a complementary loss-of-function approach in which we transfected A20/D1.3 cells with a combination of ERK1- and ERK2-specific siRNAs. The combined ERK1/2 siRNA transfection reduced the levels of ERK1/2 in the cells by ~68% compared to cells transfected with control non-targeting siRNA (Figure 1C). Importantly, siRNA-mediated depletion of ERK1 and ERK2 significantly reduced the ability of the cells to spread on coverslips coated with either 0.625 µg/cm ^2^ or 1.25 µg/cm ^2^ anti-IgG (Figure 1D and 1E).

Finally, we showed that pre-treating A20/D1.3 cells with either FR180204 or U0126 reduced their ability to spread on coverslips coated with HEL (Figure 1F and 1G). As was the case for spreading on anti-IgG, the ERK inhibitor was more effective than the MEK inhibitor at reducing B cell spreading on HEL, and greater reductions in spreading area were observed at the lower density of HEL (0.22 µg/cm ^2^ ) than at a higher density (0.44 µg/cm ^2^ ). Hence, ERK activity contributes to both antigen- and anti-IgG-induced B cell spreading, especially at lower densities of these activating stimuli.

Although ERK has important roles in B cell development, survival, proliferation, and transcriptional regulation (Richards et al., 2001; Yasuda et al., 2008; O'Reilly et al., 2009), to our knowledge this is the first report that ERK is involved in BCR-induced spreading. How ERK regulates the actin remodeling that drives B cell spreading remains to be determined. The radial spreading of B cells on antigen-coated surfaces is driven by branched actin polymerization nucleated by the Arp2/3 complex (Bolger-Munro et al., 2019). In fibroblasts, ERK signaling recruits the Arp2/3 complex to the leading edge of motile cells and increases Arp2/3 complex-mediated actin polymerization that powers the outward growth of lamellipodial membrane protrusions (Mendoza et al., 2015). Whether ERK activity enhances Arp2/3 complex recruitment or activity at the peripheral actin ring of spreading B cells remains to be assessed. The Arp2/3 complex is activated by the WASp, N-WASp, and WAVE2 nucleation-promoting factors (Rottner et al., 2017). It had been reported that ERK promotes the formation of lamellipodia by phosphorylating WAVE2 and its binding partner Abi1 (Danson et al., 2007; Nakanishi et al., 2007; Mendoza et al., 2011; Mendoza, 2013). However, a recent study found that these putative ERK phosphorylation sites are not important for lamellipodia formation or cell motility in several cell types and that WAVE2 phosphorylation is ERK-independent (Singh et al., 2020). A number of other actin-regulatory proteins such as cortactin, filamin A, and paxillin are putative substrates of either ERK or the p90Rsk and Mnk1 kinases that are activated by ERK (Tanimura and Takeda, 2017; Lavoie et al., 2020). Phosphoproteomic analyses of control versus ERK-inhibited B cells spreading on immobilized antigen could identify candidate ERK effectors whose role in linking BCR engagement to actin remodeling could then be studied.

Dynamic reorganization of the actin cytoskeleton is especially important for B cells to respond to antigens that are displayed on the surface of APCs (Bolger-Munro et al., 2019). In this case, actin-dependent formation of an immune synapse amplifies BCR signaling and may lower the threshold for the amount of cell-bound antigen required to trigger B cell activation and proliferation. Further work is needed to test whether ERK activity is important for APC-induced B cell activation. B cell activation is also dependent on actin-dependent cell motility that enables B cells to migrate into and within lymphoid organs to encounter antigens and then interact with helper T cells (Cyster, 2010). Based on our findings, further studies should assess the role of ERK activity in chemokine-directed B cell migration. ERK inhibitors are currently in clinical trials for cancer therapy (Sullivan et al., 2018; Chin et al., 2019), raising the possibility that these drugs could be used to reduce aberrant B cell activation in autoimmune diseases.

## Methods

**Cell culture**

The A20 murine IgG+ B lymphoma cell line (Kim et al., 1979) was obtained from ATCC (#TIB-208). The A20/D1.3 murine B cell line (Batista and Neuberger, 1998), which expresses a transfected HEL-specific IgM-containing BCR in addition to its endogenous IgG-containing BCR, was a gift from F. Batista (Ragon Institute, Cambridge, MA). The cell lines were confirmed to be mycoplasma-negative and were cultured in RPMI-1640 (Sigma, #R0883) supplemented with 5% heat-inactivated fetal bovine serum (FBS) (Gibco, #12483020), 2 mM glutamine (Fisher Scientific, #O2956-100), 1 mM pyruvate (Gibco, #11360070), 50 μM 2-mercaptoethanol (Sigma-Aldrich, #M3148), 50 U/mL penicillin, and 50 μg/mL streptomycin (Gibco, #15140122).

**Chemical inhibitors**

The ERK inhibitor FR180204 (Sigma-Aldrich, #SML0320) (Tocris Bioscience, #3706) (Ohori et al., 2005) and the MEK inhibitor U0126 (Cayman Chemical, #70970) (Favata et al., 1998) were used at final concentrations of 30 µM.

**siRNA transfection and immunoblotting**

Using an Amaxa Nucleofector (program L-013) and the Ingenio Electroporation Kit (Mirus, #MIR50118), 3 x 10 ^6^ A20/D1.3 cells were transiently transfected with 4 µg of control non-targeting siRNA (Dharmacon, #D-001810-10-20) or with 4 µg each of mouse MAPK3/ERK1-specific (Dharmacon, #L-040126-00-0005) and mouse MAPK1/ERK2-specific siRNA (Dharmacon, #L-040613-00-0005). The cells were then cultured for 24 hr prior to use. siRNA-mediated depletion of ERK1 and ERK2 was analyzed by immunoblotting. Cell extracts were prepared, separated on 12% SDS-PAGE gels, and analyzed by immunoblotting as described previously (Bolger-Munro et al., 2021). Nitrocellulose membranes were incubated with a rabbit anti-ERK1/2 antibody (Cell Signaling Technologies, #9102, 1:1000) followed by horseradish peroxidase-conjugated goat anti-rabbit IgG (Bio-Rad, #170-6515, 1:3000) or with a mouse anti-β-actin antibody (Santa Cruz, #Sc-47778, 1:3000) followed by horseradish peroxidase-conjugated goat anti-mouse IgG (Bio-Rad, #170-6516, 1:3000). Immunoreactive bands were visualized using an enhanced chemiluminescence detection reagent (Azure Biosystems, #AC2010) and quantified using a Li-Cor C-DiGit Scanner and Image Studio software. ERK signals were normalized to the actin band intensity for the same sample.

**Cell spreading assays and cell area quantification**

Cell spreading assays were performed as described previously (Bolger-Munro et al., 2021). Glass coverslips were coated with the indicated amounts of goat anti-mouse IgG (Jackson ImmunoResearch, #115-005-008) or HEL (NANOCS, #LSN-BN-1) for 1 hr at room temperature and then blocked with 2% bovine serum albumin (BSA) in PBS for 30 min at room temperature. A20 or A20/D1.3 cells were resuspended to 7.5 x 10 ^5^ /mL in modified HEPES-buffered saline (25 mM sodium HEPES pH 7.4, 125 mM NaCl, 5 mM KCl, 1 mM CaCl _2_ , 1 mM Na _2_ HPO _4,_ 0.5 mM MgSO _4_ , 1 g/L glucose, 2 mM glutamine, 1 mM sodium pyruvate, 50 μM 2-mercaptoethanol) with 2% FBS. The cells were pre-treated with the ERK or MEK inhibitor, or an equivalent volume of DMSO, for 1 hr at 37 ^o^ C before adding 7.5 x 10 ^4^ cells in 100 µL to each coverslip. After the indicated times at 37 ^o^ C, the cells were fixed by adding 100 µL of 8% PFA (4% final concentration) for 10 min and then permeabilized with 0.2% Triton X-100 in PBS for 5 min at room temperature. F-actin was visualized by staining with rhodamine-conjugated phalloidin (Thermo Fisher, #R415, 1:400 in dilution in PBS) for 30 min at room temperature. Coverslips were mounted onto slides using ProLong Diamond anti-fade reagent (Thermo Fisher, #P36965). Images of the B cell-coverslip interface were captured using a Zeiss Axiovert 200M spinning disk confocal microscope with a 100X NA 1.45 oil objective lens. The cell area was quantified from thresholded binary images using Fiji software (Schindelin et al., 2012). The outer face of the peripheral actin ring was used to define the cell edge and compute the total cell area.

**Statistical Analysis**

For individual cell spreading experiments, the data are presented as dot plots and the Mann-Whitney U test was used to compare ranked values between the samples. Two-tailed paired t-tests were used to compare mean values for matched sets of samples from multiple experiments.
